# Development of a porcine acellular bladder matrix for tissue-engineered bladder reconstruction

**DOI:** 10.1007/s00383-022-05094-2

**Published:** 2022-03-22

**Authors:** Massimo Garriboli, Koichi Deguchi, Giorgia Totonelli, Fanourios Georgiades, Luca Urbani, Marco Ghionzoli, Alan J. Burns, Neil J. Sebire, Mark Turmaine, Simon Eaton, Paolo De Coppi

**Affiliations:** 1 Stem Cells and Regenerative Medicine Section, Developmental Biology and Cancer Programme UCL Great Ormond Street Institute of Child Health, 30 Guilford Street, London, WC1N 1EH UK; 2 Department of Nephro-Urology, Evelina London Children’s Hospital, Guys and St. Thomas NHS Foundation Trust, London, UK; 3 Department of Pediatric Surgery, Graduate School of Medicine, Osaka University, Suita, Osaka Japan; 4 Neural Development Unit, Institute of Child Health, University College London, 30 Guilford Street, London, UK; 5 Department of Histopathology, Institute of Child Health and Great Ormond Street Hospital, University College London, London, UK; 6 Division of Bioscience, University College London, London, UK; 7 Paediatric Surgery Department, Great Ormond Street Hospital, London, UK

**Keywords:** Bladder augmentation, De-cellularisation, Extracellular matrix, Tissue engineering

## Abstract

**Purpose:**

Enterocystoplasty is adopted for patients requiring bladder augmentation, but significant long-term complications highlight need for alternatives. We established a protocol for creating a natural-derived bladder extracellular matrix (BEM) for developing tissue-engineered bladder, and investigated its structural and functional characteristics.

**Methods:**

Porcine bladders were de-cellularised with a dynamic detergent–enzymatic treatment using peristaltic infusion. Samples and fresh controls were evaluated using histological staining, ultrastructure (electron microscopy), collagen, glycosaminoglycans and DNA quantification and biomechanical testing. Compliance and angiogenic properties (Chicken chorioallantoic membrane [CAM] assay) were evaluated. *T* test compared stiffness and glycosaminoglycans, collagen and DNA quantity. *p* value of < 0.05 was regarded as significant.

**Results:**

Histological evaluation demonstrated absence of cells with preservation of tissue matrix architecture (collagen and elastin). DNA was 0.01 μg/mg, significantly reduced compared to fresh tissue 0.13 μg/mg (*p* < 0.01). BEM had increased tensile strength (0.259 ± 0.022 vs 0.116 ± 0.006, respectively, *p* < 0.0001) and stiffness (0.00075 ± 0.00016 vs 0.00726 ± 0.00216, *p* = 0.011). CAM assay showed significantly increased number of convergent allantoic vessels after 6 days compared to day 1 (*p* < 0.01). Urodynamic studies showed that BEM maintains or increases capacity and compliance.

**Conclusion:**

Dynamic detergent–enzymatic treatment produces a BEM which retains structural characteristics, increases strength and stiffness and is more compliant than native tissue. Furthermore, BEM shows angiogenic potential. These data suggest the use of BEM for development of tissue-engineered bladder for patients requiring bladder augmentation.

## Introduction

Both congenital and acquired pathologies can result in loss of bladder capacity or function [1]. For end-stage bladder disease, the most commonly performed surgical procedure is enterocystoplasty, which is associated with complications and long-term risk of malignancy [2–4]. Alternative strategies may include tissue engineering, where bladder replacement using synthetic scaffolds has been obtained in both experimental models and in a limited number of patients [5–7]. Synthetic scaffolds may however be limited on mimicking all fine characteristics of the original organ. Instead, de-cellularised matrices maintain the organ microarchitecture, including native vasculature, along with bio-factors needed for cell growth and repopulation into the extracellular matrix (ECM) whilst remaining non-immunogenic. They have been successfully utilised in human for simple tubular structures such as the trachea [8], and experimentally for more complex organs, such as the oesophagus [9, 10], small intestine [9], heart [11] and lung [12].

The bladder consists of an inner urothelium and an outer smooth musculo-vascular layer, connected and intimately joined by bladder extracellular matrix (BEM) composed of collagens (type I and III) and elastic fibres. Bladder acellular matrices obtained via static enzymatic treatment have been described [13], but the best approach that provides a complete removal of the cellular component while maintaining the inherent characteristics of the bladder wall has yet to be described.

Our aim was to generate an acellular natural matrix from porcine bladder that maintains architectural and structural properties whilst removing cells and immunogenicity, thus could represent a substrate for seeding human urothelial cells and inducing maturation into a functional urothelial layer for tissue engineering bladder augmentation.

## Materials and methods

### Organ harvest

All surgical procedures and animal husbandry were in accordance with UK Home Office guidelines under the Animals (Scientific Procedures) Act 1986. Bladders from young pigs (30 kg) were obtained at the Royal Veterinary College.

### Detergent-enzymatic treatment (DET)

After washing and clearing of peri-vesical fat, a 12-Fr trans-urethral catheter was inserted and organs either perfused continuously at 10 mL/h (dynamic de-cellularisation) or filled with 500 mL of each solution (static de-cellularisation). The DET cycle was composed of de-ionised water (resistivity 18.2 MΩ/cm) at 4 °C for 24 h, 4% sodium deoxycholate (Sigma) at room temperature (RT) for 4 h, and 2000 kU DNase-I (Sigma) in 1 M NaCl (Sigma) at RT for three hours, as previously described [9]. After the cycle of treatment, the constructs were preserved at 4 °C, in phosphate-buffered saline with antibiotic–anti-mycotic (PBS/AA).

### DNA quantification

Specimens were disintegrated and homogenised in 1 mL lysis buffer (50 mM Tris–HCl [pH 8], 50 mM EDTA, 1% SDS and 10 mM NaCl). Samples were digested with Proteinase K overnight, followed by phenol/chloroform extraction. DNA was precipitated from the aqueous phase with 100% ethanol and washed with 70% ethanol. The pellet was dissolved in ribonuclease-free water, stored at − 20 °C and purity (260 and 280 nm) and yield (280 nm) of nucleic acids were quantified spectrophotometrically.

### Histological analysis

Samples were formalin-fixed, paraffin-embedded and sectioned at 4 μm. Sections were stained with Haematoxylin and Eosin (H&E) (Leica), Masson trichrome (MT), (Leica, Raymond A Lamb, BDH Chemicals Ltd), Elastica van Gieson (EVG) (VWR, Leica, Raymond A Lamb), and Picrosirius red (PR) staining.

### Collagen quantification

Collagen content of fresh and de-cellularised bladders was quantified using the QuickZyme total collagen assay (Biosciences) according to the manufacturer’s instructions. Briefly, the samples were digested overnight in 6 M HCl at 95 °C. Extracts were diluted with 4 M HCl and incubated with assay buffer for 20 min at RT. Detection reagents were added and incubated for 1 h at 60 °C. Absorbance was determined at 570 nm with a microplate reader. Collagen concentrations from a standard curve were used to calculate the collagen content of the tissue.

### Glycosaminoglycan quantification

The sulfated glycosaminoglycan (GAG) content of fresh and de-cellularised bladders was quantified using the Blyscan GAG Assay Kit (Biocolor, UK). In brief, 50 mg of minced wet tissue was weighed and placed in a micro-centrifuge tube containing 1 mL of Papain digestion buffer and incubated at 65 °C for 18 h, with occasional tube removal and vortexing. Aliquots of each sample were mixed with 1,9-dimethyl-methylene blue dye and reagents from the GAG assay kit. The absorbance at 595 nm was measured using a microplate reader and compared to standards made from bovine tracheal chondroitin-4-sulfate to determine absolute GAG content.

### Biomechanical tests

Specimens were subjected to uniaxial tension until failure, which records the tensile strength “s” (Stress) versus strain “ε”; the highest point of the stress–strain curve is the Ultimate Tensile Strength (UTS). The ratio of stress to strain is the Young’s modulus E, which is a measure of the stiffness of an elastic material. Mechanical tests were performed with the application of uniaxial tension in an Instron 5565 at room temperature (20 ± 1 °C). Specimens in the form of flat dumbbells with a 20 mm long working part were loaded at a constant tension rate of 100 mm/min. The thickness of the samples was measured using a digital electronic micrometer (RS components) at three places of the dumbbell and averaged. Stress–strain relationships, ultimate tensile strength (UTS), defined as maximum stress that a material could withstand until it breaks, and tensile modulus were obtained for samples and graphs plotted. Five samples harvested from different part of the organs were considered for each evaluated tissue.

### Pressure/volume curve

To evaluate compliance of the scaffold obtained after the de-cellularisation treatment, a pressure/volume curve measurement was performed. Briefly, a 12-Fr Foley catheter was inserted and the ureters closed with a 6–0 Polypropylene suture. The catheter was then connected to a 50 mL bottle held at a height of 5 cm. The bladder was then filled with water by gravity until the internal pressure reached 5 cm water. This was then repeated stepwise up to 40 cm H_2_O or the maximum bladder capacity.

### Scanning electron microscopy (SEM)

Samples were fixed in 2% glutaraldehyde in 0.1 M phosphate buffer for 24 h at 4 °C. Following washing with 0.1 M phosphate buffer, they were cut into segments of approximately 1 cm and cryoprotected in 25% sucrose, 10% glycerol in 0.05 M PBS (pH 7.4) for 2 h, then fast-frozen in Nitrogen slush and fractured at approximately − 160 °C. After washing in 0.1 M phosphate buffer (pH 7.4), the material was fixed in 1% OsO4/0.1 M phosphate buffer (pH 7.3) at 3 °C for 11/2 h and washed again in 0.1 M phosphate buffer (pH 7.4).

After rinsing with dH_2_O, specimens were dehydrated in a graded ethanol–water series, critical point dried using CO_2_ and finally mounted on aluminium stubs using sticky carbon taps. The fractured material was mounted to present fractured surfaces across the lumen wall to the beam. The complete samples were opened and mounted to show the lumen surface, then coated with a thin layer of Au/Pd (approximately 2 nm thick) using a Gatan ion beam coater. Images were recorded with a Jeol 7401 FEG scanning electron microscope.

### Transmission electron microscopy (TEM)

Samples were cut into segments approximately 1 cm in length. After washing in 0.1 M phosphate buffer (pH 7.4), they were fixed in 1% OsO4/0.1 M phosphate buffer (pH 7.3) at 4 °C for 11/2 h then washed in 0.1 M phosphate buffer (pH 7.4). Specimens were stained en bloc with 0.5% uranyl acetate dH_2_O at 4 °C for 30 min, rinsed with dH_2_O, dehydrated in a graded ethanol–water series and infiltrated with Agar 100 resin and then hardened. 1 mm sections were cut and stained with 1% toluidine blue in dH_2_O for light microscopy. A representative area was selected and sections were cut at 70–80 nm using a diamond knife on a Reichert ultra-cut E microtome. Sections were collected on 200-mesh copper, coated slot grid and stained with uranyl acetate and lead citrate. Images were recorded with a Jeol 1010 transition electron microscope.

### Chicken chorioallantoic membrane (CAM) angiogenic assay

To evaluate angiogenic properties of de-cellularised tissue in vivo, we used the CAM assay as previously described [9]. Briefly, at day eight of incubation, 1 mm diameter BEM and polyester (negative control) were placed on the CAM between branches of the blood vessels. Samples were examined daily until 6 days after placement when they were photographed in ovo with a stereomicroscope (Leica). The number of blood vessels < 10 μm in diameter converging towards the placed tissues was counted blindly by assessors (*n* = 5), with the mean of the counts being considered.

## Results

### Macroscopic appearance

**Fig. 1:**
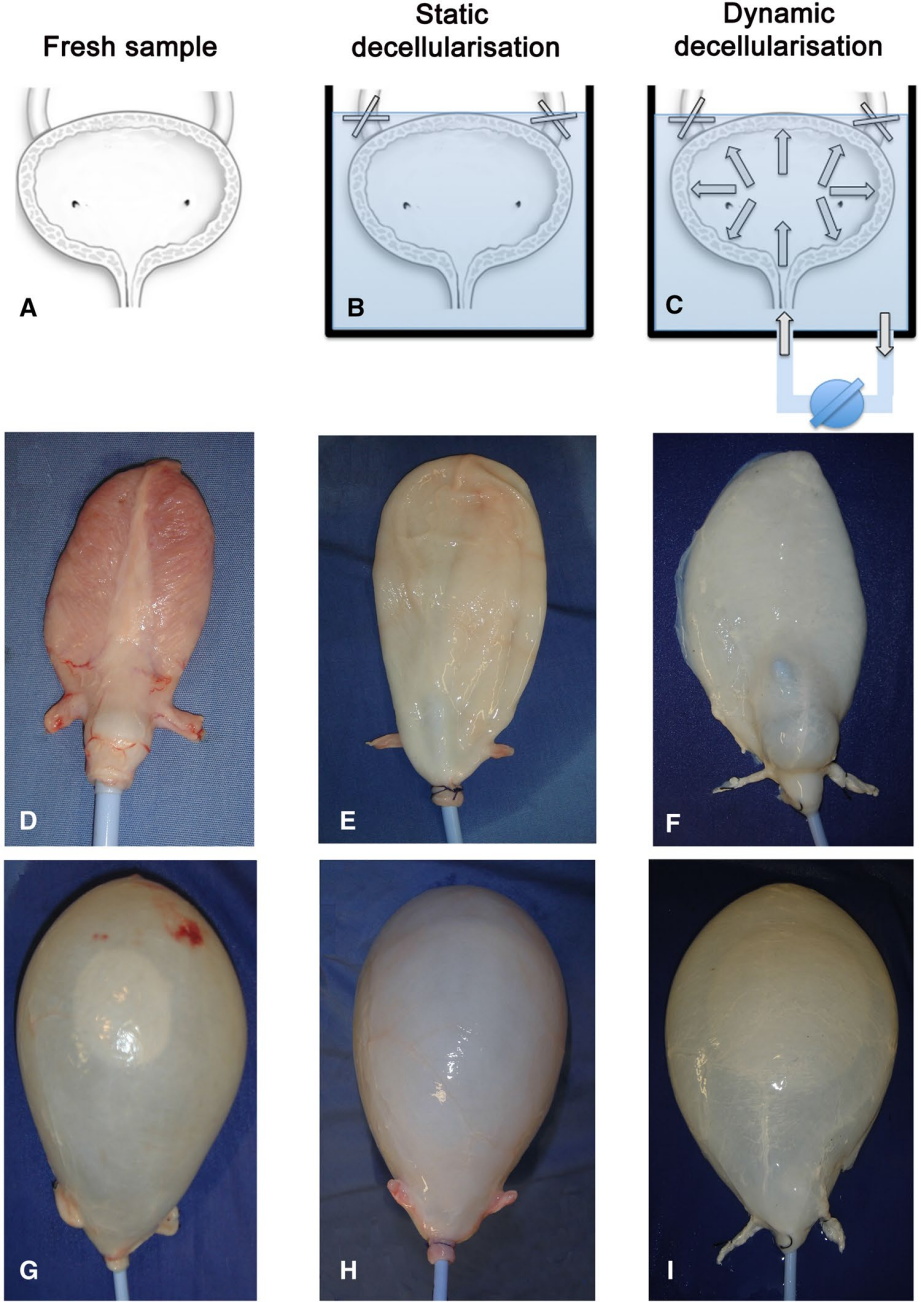
Fresh bladder samples (**A**) underwent static (**B**) and dynamic (**C**) decellularisation of porcine bladder with detergent-enzymatic treatment. Macroscopic images of fresh bladder empty (**D**) and filled with 500 mL of saline (**G**). Bladder following one cycle of static decellularisation empty (**E**) and filled with 500 mL of saline (**H**). Bladder after dynamic decellularisation before (**F**) and after filling with 500 mL of saline (**I**)

Whole bladders harvested from adult pigs were either maintained in static condition or perfused dynamically with DET (Fig. 1). Macroscopically, treated bladders maintained the overall morphology when compared to the fresh sample. However, the bladder wall in the group treated with static de-cellularisation did partially loose the natural pinkish appearance (Fig. 1E, H), whereas the group treated with dynamic perfusion assumed a white/transparent feature (Fig. 1F, I). In both groups, the macroscopic appearance of the trigone was maintained after the treatment leaving the relationship between ureters, bladder outlet and bladder neck intact (data not shown).

### DNA quantification

**Fig. 2:**
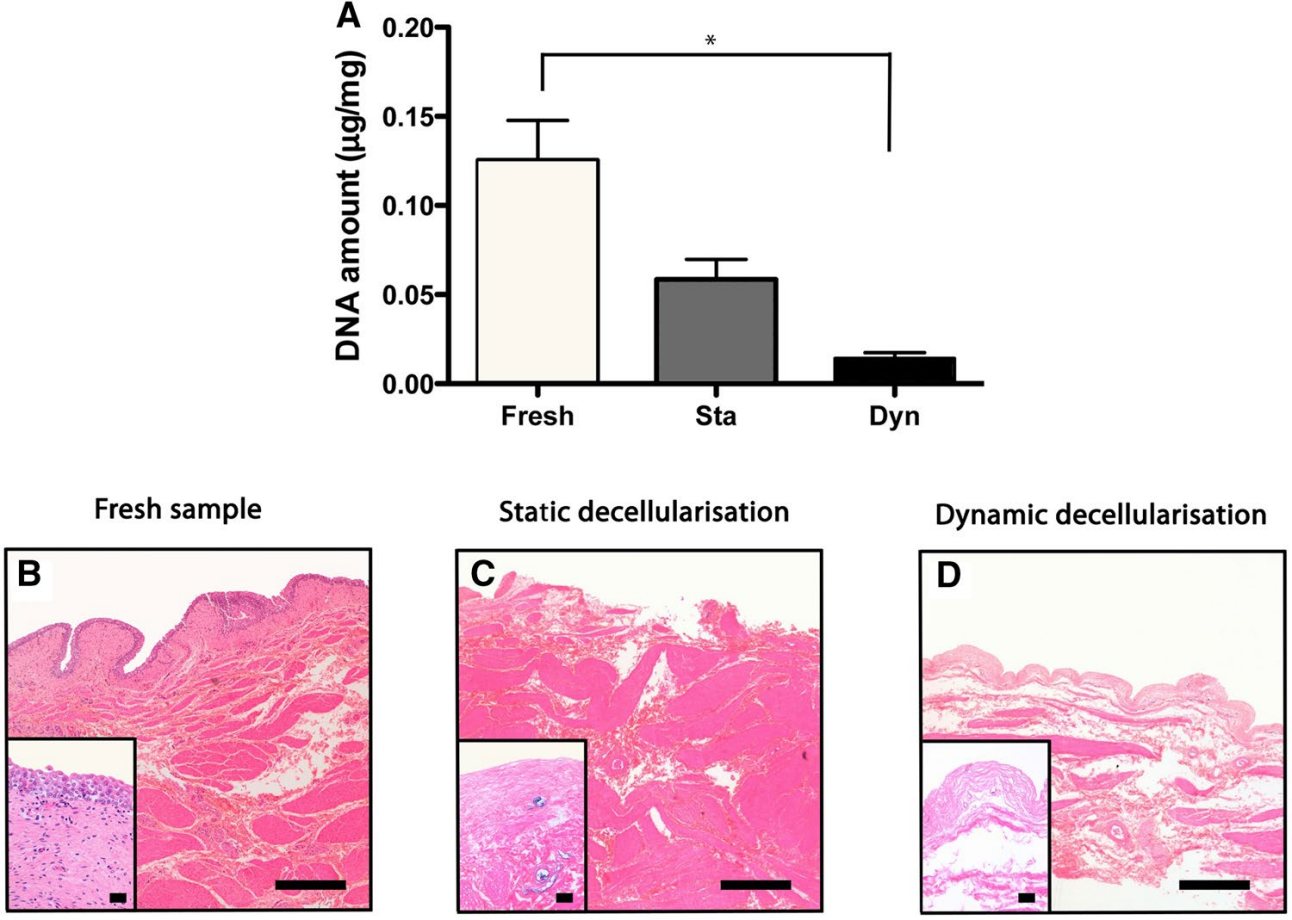
DNA quantification shows significant reduction of DNA following 1 cycle of dynamic detergent-enzymatic treatment (*p < 0.001) whereas the group treated with static decellularisation did not show a significant reduction when compared to fresh controls (**A**). Haematoxylin and Eosin (H&E) staining confirms the absence of nuclei after dynamic decellularisation (**D**) and a marked reduction after static decellularisation (**C**). After both treatments, the structure is preserved compared to fresh tissue (**B**–**D**). Scale bar: 50 μm

Interestingly, DNA assay showed significant reduction in DNA content after 1 cycle of dynamic de-cellularization (0.01 ± 0.006 μg/mg) compared with fresh bladder (0.13 ± 0.036 μg/mg, *p* < 0.0001), whereas the group treated with static de-cellularisation did not show a significant reduction (0.05 ± 0.022 μg/mg, *p* > 0.05, Fig. 2A).

### Histological analysis

**Fig. 3:**
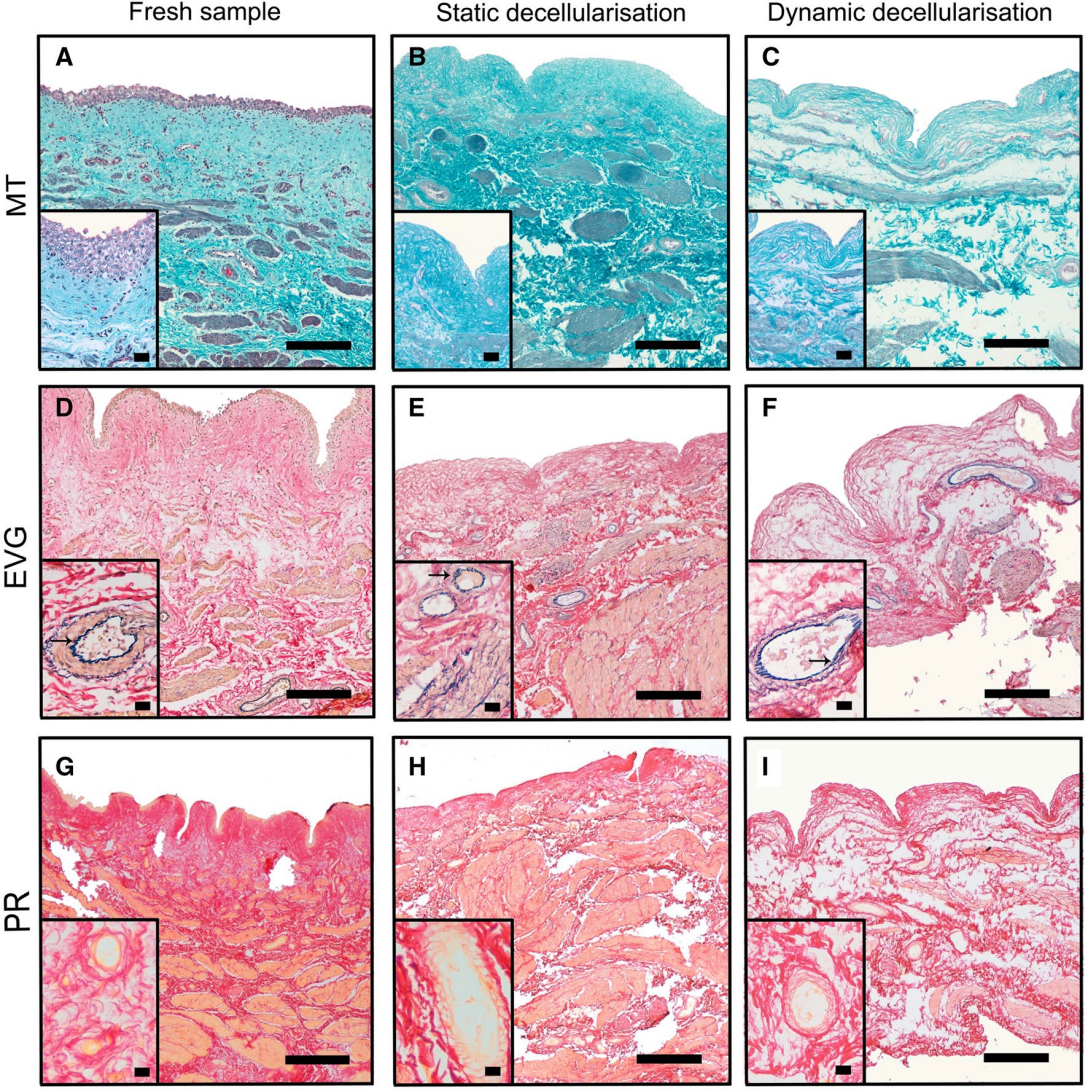
Masson trichrome (MT) staining confirmed that both decellularisation methods induced an almost complete removal of cellular (purple) and cytoplasmic material (pink) together with preservation of collagen (blue) across the wall (**A**–**C**). Elastica Van Gieson (EVG; **D**–**F**) and Picrosirius Red (PR; **G**–**I**) staining exhibit, respectively, preservation of elastin and collagen, respectively. Arrows indicate fibres of elastin staining blue around the blood vessels in both fresh and decellularised specimens. Scale bar: 50 μm

H&E showed, in both static and dynamic decellularised bladders, the absence of nuclei whilst maintaining the architecture of the mucosa submucosa and muscularis propria (Fig. 2B–D). MT staining confirmed in both groups the almost complete removal of cellular (purple) and cytoplasmic material (pink) together with preservation of collagen (blue) across the wall (Fig. 3A–C). Similarly, EVG (Fig. 3D–F) and PR (Fig. 3G–I) staining exhibit, respectively, preservation of elastin and collagen after one cycle of DET. In particular, fibres of elastin staining blue are evident all around the blood vessels in both fresh and decellularised specimens (see arrows in Fig. 3D–F).

Considering these findings, we decided to abandon the static treatment and to continue only with BEM obtained with dynamic DET, which were fully characterised using further morphological and functional tests.

### Collagen and Glycosaminoglycan quantification

**Fig. 4:**
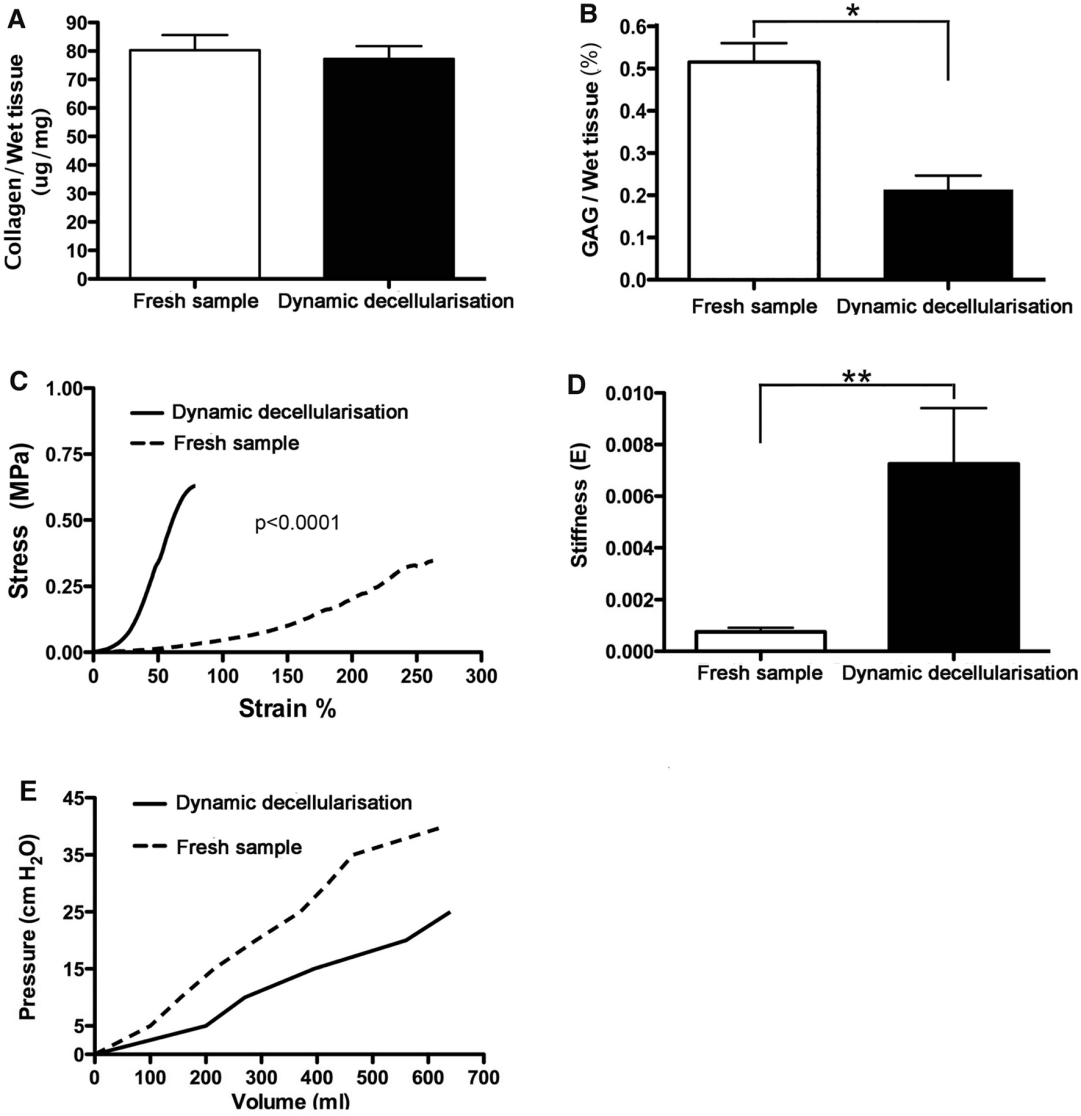
Analysis of dynamic decellularised bladders. Collagen content (**A**) was unchanged after treatment (*p* = 0.68); sulfated glycosaminoglycan (GAG) was significantly (*p = 0.006) depleted after decellularisation (**B**). Mechanical characterization of the acellular matrix: stress–strain curves show the tensile strength and stiffness significantly increased in bladder extracellular matrix (BEM) when compared with fresh tissue (**C**, **D**; **p = 0.0113). Pressure–Volume curves are linear both in fresh and decellularised bladders, and showed that BEM were more compliant compared with fresh bladder (**E**)

Histological findings were confirmed using quantitative parameters. Collagen content was unchanged (Fig. 4A, *p* = 0.68) after treatment but GAG was significantly depleted by decellularisation (Fig. 4B, *p* = 0.006).

### Biomechanical tests

Tensile strength was higher in BEM compared with fresh tissue (0.259 ± 0.022 vs. 0.116 ± 0.006, respectively, *p* < 0.0001) (Fig. 4C, D). Stiffness was also significantly increased in BEM when compared to controls (0.00075 ± 0.00016 vs. 0.00726 ± 0.00216), (*p* = 0.0113).

### Pressure–volume curve

Pressure–Volume curves were linear and showed that the BEM was more compliant compared with fresh bladder accommodating more volume before increasing internal pressure, e.g. at 25 cmH_2_O, fresh bladder filled with an average of 338 mL while the BEM accommodated 620 mL. Above that volume, in the BEM samples, instead of increasing the internal pressure we noted the water exuding throughout the wall (Fig. 4E).

### Scanning and transmission electron microscopy (SEM and TEM)

**Fig. 5:**
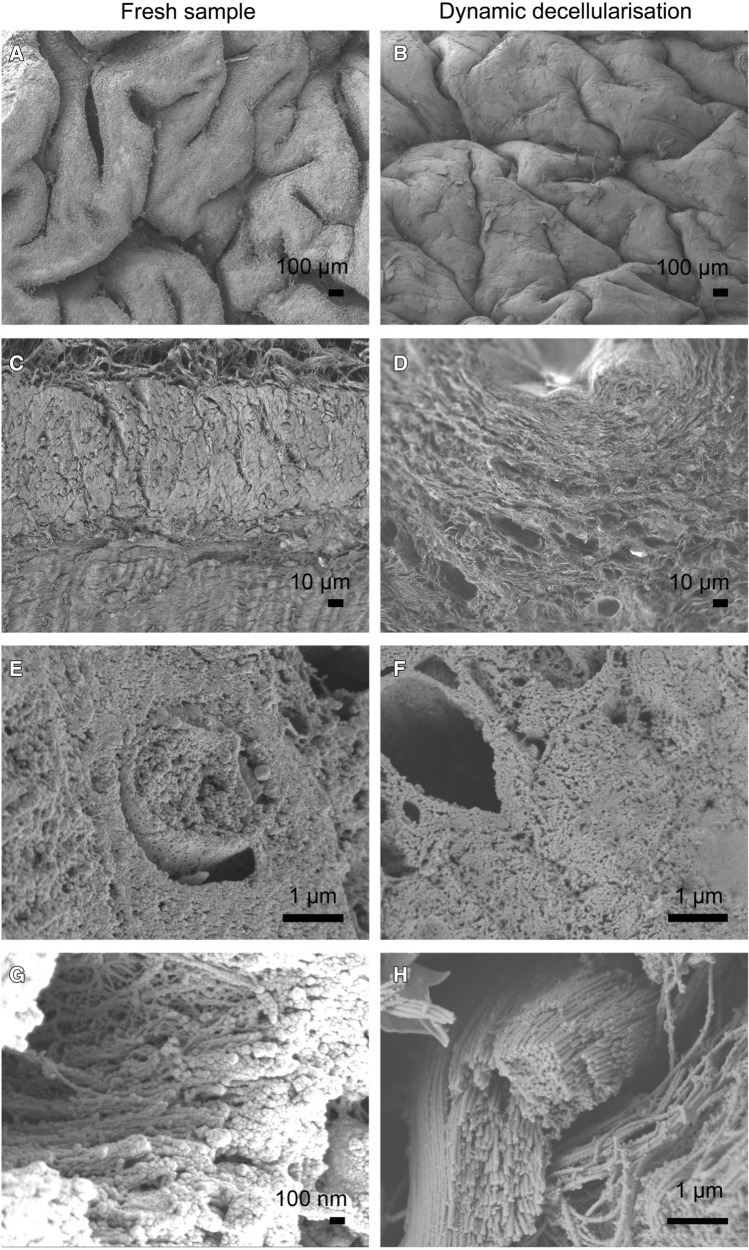
SEM (scanning electron microscopy) of fresh bladder (**A**, **C**, **E**, **G**) and after 1 cycle of decellularisation (**B**, **D**, **F**, **H**). Ultra-structural characteristics of native tissue are preserved while viable cells are completely absent

**Fig. 6:**
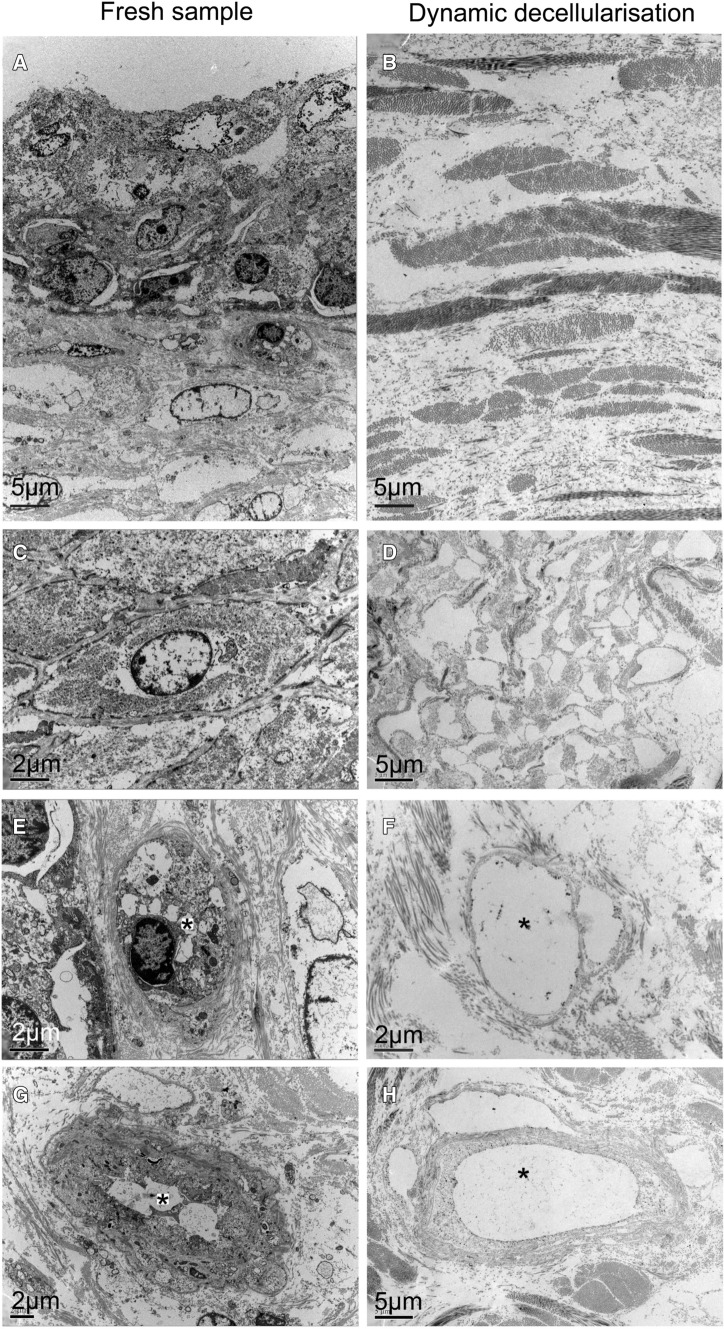
TEM (transmission electron microscopy) of fresh bladder (**A**, **C**, **E**, **G**) and after 1 cycle of decellularization (**B**, **D**, **F**, **H**). Microstructure is maintained in decellularised samples (* indicate capillary lumen)

SEM of the BEM compared with fresh bladder showed preservation of the micro- and ultra-structural characteristics of native tissue and confirmed the absence of viable cells (Fig. 5). In particular, the luminal surface after DET revealed a flat surface with the same macroscopic features compared with the fresh control in which, however, the surface was irregular due to the presence of urothelial cells (Fig. 5A, B). Wall section of the BEM showed a network of collagen fibres more tightly compacted compared to the fresh sample; however, the extracellular microstructure of the bladder wall was maintained (Fig. 5C–H). TEM confirmed the absence of cells in the decellularised scaffold with highly preserved fibrillar structure (Fig. 6).

### Chicken chorioallantoic membrane (CAM) angiogenic assay

**Fig. 7:**
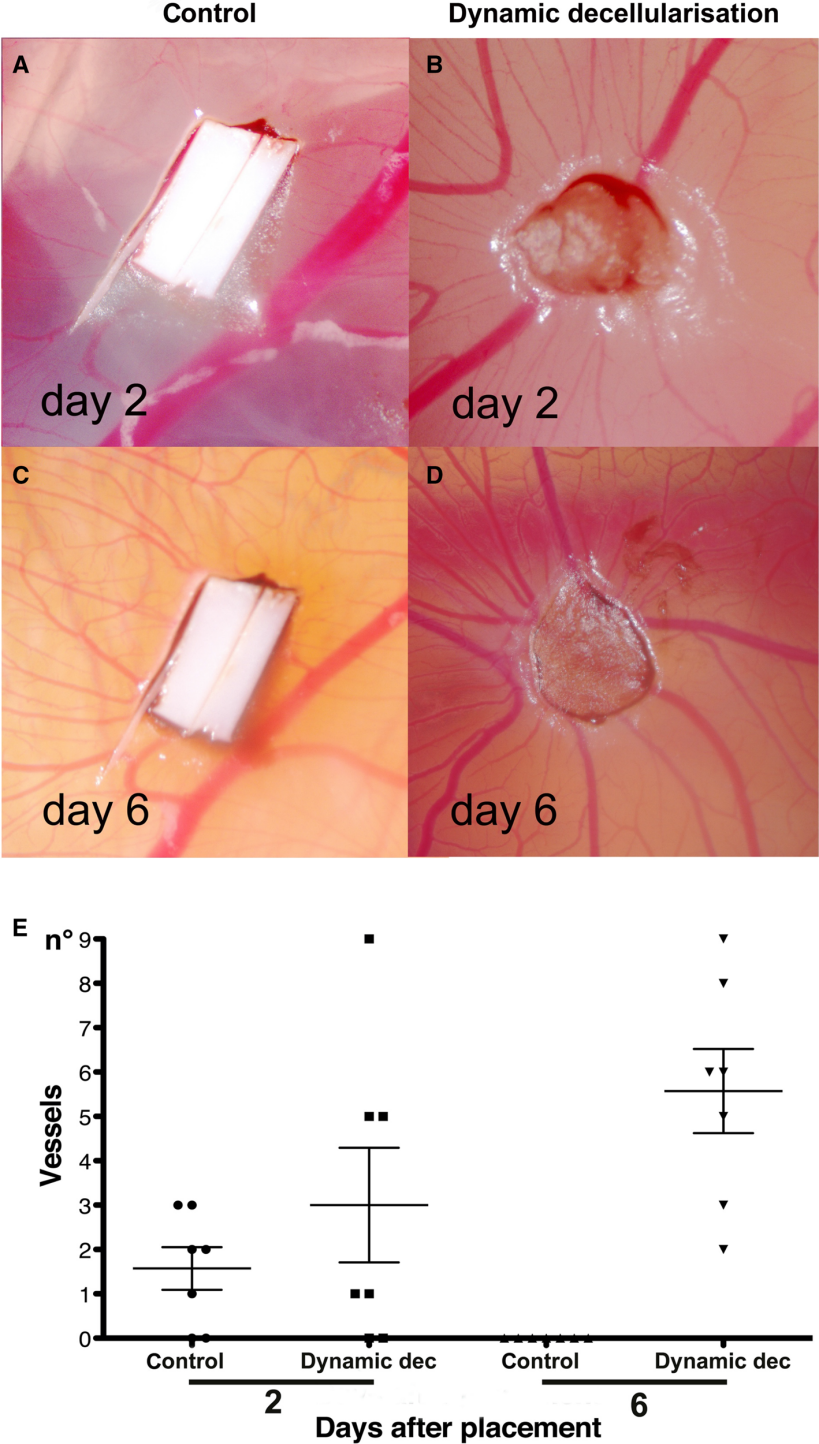
Pro-angiogenic properties of BEM in vivo (**B**, **D**). Macroscopic quantification of converging vessels was blindly made for both decellularised bladder samples and polyester membrane used as negative control (**A**, **C**). On day six after implantation, the number of vessels converging towards the BEM is significantly increased in comparison to the same samples at day two and to the polyester membrane (**E**; *p* < 0.05)

To test the ability of the BEM to attract blood vessels, we used an established in in vivo system [9, 14, 15] where tissue samples were placed on the chicken egg CAM. Decellularised bladder and polyester membrane controls were analysed daily under a stereomicroscope. After two days, BEM was adherent to the CAM and started to be surrounded by allantoic vessels (Fig. 7B). Six days following implantation, BEM was enveloped by newly formed vessels, which did orientate in a “spoked wheel” pattern (Fig. 7D). Macroscopic quantification of converging vessels was made blindly for both BEM samples and controls. The number of allantoic vessels converging towards the BEM was significantly increased compared between day two and six, and was also significantly greater on day six between BEM and control (Fig. 7E, *p* < 0.05).

## Discussion

The present work shows that using an innovative perfusion technique, an acellular natural matrix can be obtained from porcine bladder without depletion of structural and mechanical characteristics of the native tissue and can represent a valid substrate for culturing and expanding urothelial cells. This has important implications for clinical delivery of tissue-engineered bladder for patients requiring bladder augmentation or substitution.

In the last two decades, tissue-engineered extracellular matrices (ECMs) [16, 17] and artificial polymer-based constructs have been experimentally utilised for bladder reconstruction [18, 19] but without functional outcome comparable to current techniques [20–22]. Despite some high-profile reports [7], in fact, critical evaluation of the literature reveals little evidence that bladder tissue engineering can yet achieve a functional outcome equivalent to current surgical techniques [23] and thus there remains an unmet clinical need [24].

Pioneering work in bladder tissue engineering used Polyglycolic acid (PGA) constructs in dogs [25] and in a small series of patients with neuropathic bladder [7]. This approach utilised autologous bladder urothelial and muscle cells, expanded in in vitro, and attached to a composite biodegradable PGA scaffold wrapped in omentum to provide a vascular support to the transplanted cells. However, although promising, the results obtained with patients have not been followed by successful translation into a phase 2 human trial [26].

Tissue engineering techniques for bladder regeneration are challenged by pressure changes associated with filling and voiding cycles, so that any biomaterial used in bladder reconstruction must provide suitable structural properties. Although both ECMs and polymer-based constructs have potential advantages over autologous gastrointestinal tissue, ECMs preserve structure and composition of the original organ and therefore may offer more consistent long-term results [5].

Several protocols to create a BEM have been published that mainly differ based on the type of decellularisation technique (physical [27], mechanical [28] or chemical [29]). The novelty of our protocol is that it uses both a gently mechanical force obtained through the continuous perfusion and the detergent-enzymatic treatment. We have demonstrated that the peristaltic infusion contributes to a more efficient removal of the cellular component (Fig. 2) without adding the risk to compromise the quality of the extracellular matrix.

As alternative to BEM, some authors have used seeded or unseeded small intestinal submucosa (SIS). Experimentally, when substituted for a small part of the organ good results were obtained [30, 31] but this was not confirmed in a subtotal cystectomy model [32]. Furthermore, the use of SIS has been demonstrated to be unsuccessful in human series performed by Caione et al*.* [33] and Schaefer et al*.* [32]. More recently Zhang et al*.*, presented a small human series, consisted of 15 neurogenic bladder patients augmented with SIS, and suggested that bladder augmentation with SIS graft cannot be recommended in patients with seriously damaged bladder and severe vesico-ureteric reflux [34].

To solve the need for bladder augmentation, an approach alternative to the use of scaffolds, has been proposed by Southgate et al. when they described a composite cystoplasty, using autologous urothelial cell sheets grown and expanded in vitro combined with a host pedicled and de-epithelialised smooth muscle segment, such as uterus or intestine [35, 36]. Similarly, the group from the University of California presented a porcine model of reconstruction obtained through the use of a demucosalized colonic segment repopulated through aerosol transfer of urothelial and smooth muscle cells [37]. Chen et al*.* also presented a similar approach using autologous tissue-engineered peritoneal grafts consisting in the peritoneal sheet and the sero-muscular segment of small intestine [38]. Those strategies may have the advantage, over a full tissue-engineered approach, that the in vitro component of the procedure is confined to propagation of a single, highly regenerative cell type. This interesting approach, has been performed in pig but has not as yet, been followed by clinical translation.

Comparing the techniques, the use of naturally derived scaffolds could diminish morbidity associated with Southgate’s approach (which still requires the use of intestine) and mimic the original tissue more closely than a synthetic scaffold (as proposed by Atala et al*.*) with regard to both mechanical properties of passive urinary bladder and whole organ mechanics [39].

Previous techniques for bladder decellularisation required harsh protocols, which failed to maintain the original structure of the tissue [40, 41] whereas our protocol generates BEM that retains structural and functional characteristics of the native bladder and would represent a natural environment for the engrafting and proliferation of human urothelial cells. Our dynamic decellularization system accelerated removal of nucleic contents; only one cycle of DET (incubation with mild detergent and DNA-degrading enzyme for seven hours in total), in fact, sufficiently removed the genetic material from the scaffold. We believe that this shortened protocol favoured the better retainment of structural and functional characteristics and minimised degradation of the ECM ultrastructure by the decellularising reagents. Diminishing incubation with decellularising reagents is also advantageous to minimise the residual cytotoxic agents such as detergent within the scaffold and therefore might facilitate the cytocompatibility of the scaffold in view of cell seeding.

Scanning and transmission electron microscopy (SEM and TEM) is often utilised for visualising the morphology of the BEM surface. SEM and TEM results provided further evidence that our dynamic decellularization protocol efficiently removed cellular components from the scaffold while retaining his ultrastructure including luminal surface and microstructure of the bladder wall, both of which are important for repopulation of urothelial cells on the luminal surface and mesenchymal cells into the bladder wall.

Vascularization is one of the key aspects for the survival of the repopulating cells in the implanted graft. The natural structure and inherent growth factors of the scaffolds affect angiogenesis of implanted graft. One of the major advantages of decellularised ECM is its retaining these structural, biomechanical, and biochemical cues, some of which can accelerate angiogenesis and improve repopulated cell survival. In the present study, significantly better neovascularisation was confirmed in the CAM assay compared to the control synthetic graft, which indicated our BEM could render a positive effect on the vascularization, therefore representing a suitable scaffold for an engineered neobladder. The use of BEM could be a simple approach to facilitate neovascularisation compared to other methods, such as the addition of growth factors, seeding of endothelial cells, or use of secretome of mesenchymal stem cells [42].

Urodynamic parameters of the tissue-engineered graft, including mechanical strength and bladder compliance, is of importance for the clinical success of bladder augmentation. Candidates for bladder augmentation had usually low compliant and high pressure bladder [43]; hence, the restoration of the bladder wall pliability using tissue-engineered bladder graft is of paramount importance. Using biomechanical tests and pressure–volume curve, we confirmed the obtained BEM preserved enough mechanical strength comparable to the fresh bladder control, while maintaining better compliance; the latter probably due to lack of the cellular component of the organ. Further study should confirm the establishment of the bladder wall compliance after re-cellularization and in vivo transplantation.

## Conclusion

We have demonstrated that the BEM obtained from porcine bladder using the proposed innovative protocol retains similar structural and functional characteristics compared to the native bladder and represent a suitable substrate for culturing urothelial cells in the process of generating functional patch for bladder tissue engineering.

## Data and material availability

The data that support the findings of this study are available from the corresponding author, PDC, upon reasonable request.
